# Prevalence and associated factors of insomnia symptoms after ending China’s dynamic zero-COVID policy: a cross-sectional survey of frontline nursing staff in Chinese hospitals

**DOI:** 10.3389/fpubh.2024.1363048

**Published:** 2024-04-02

**Authors:** Ming Zhang, Chenru Chi, Qingwei Liu, Ningying Zhou, Zhiqing Zhou, Xiubin Tao, Bin Xuan, Huan Liu

**Affiliations:** ^1^School of Educational Science, Anhui Normal University, Wuhu, Anhui, China; ^2^School of Innovation and Entrepreneurship, Wannan Medical College, Wuhu, Anhui, China; ^3^Graduate School of Wannan Medical College, Wuhu, Anhui, China; ^4^School of Nursing, Shandong First Medical University, Jinan, Shandong, China; ^5^Wuxi Maternity and Child Health Care Hospital, Wuxi School of Medicine, Jiangnan University, Wuxi, Jiangsu, China; ^6^Department of Nursing, The First Affiliated Hospital of Wannan Medical College (Yijishan Hospital of Wannan Medical College), Wuhu, Anhui, China; ^7^Department of Hemodialysis, The First Affiliated Hospital of Wannan Medical College (Yijishan Hospital of Wannan Medical College), Wuhu, Anhui, China

**Keywords:** insomnia, symptoms, COVID, policy, China

## Abstract

**Background:**

After the Chinese government announced the end of the dynamic zero-COVID policy on January 8, 2023, the COVID-19 pandemic peaked. Frontline nursing staff are at high risk of infection transmission due to their frequent contact with COVID-19 patients. In addition, due to the ending of China’s dynamic zero-COVID policy, frontline nursing staff have grappled with increased workload, fatigue, and more. This study aimed to explore the prevalence of insomnia symptoms in frontline nursing staff and its influencing factors following the end of the policy.

**Methods:**

Between January and February 2023, this study was conducted by the Wenjuanxing platform to survey frontline nursing staff in a hospital in Wuhu City, Anhui Province. All the nursing staff included in this study had a COVID-19 infection. The questionnaires included the Athens Insomnia Scale (AIS), PC-PTSD-5 Chinese Version Scale, the Fear of COVID-19 Scale, The 2-item Connor-Davidson Resilience Scale (CD-RISC-2) Scale, and the burden of COVID-19 Scale. Binary logistic regression methods were used to identify variables associated with insomnia symptoms.

**Results:**

Among the 694 frontline nursing staff, 74.5% (517/694) exhibited insomnia symptoms. Fear of COVID-19 (*p* < 0.001), the burden of COVID-19 (*p* < 0.05), PTSD (*p* < 0.001), and higher technical titles (*p* < 0.008) were highly correlated with insomnia symptoms in frontline nursing staff. Psychological resilience (*p* < 0.001) was a protective factor for insomnia symptoms among frontline nursing staff.

**Conclusion:**

After ending China’s dynamic zero-COVID policy, the prevalence of insomnia symptoms among frontline nursing staff is generally higher. This study highlights the association between insomnia symptoms and PTSD, fear of COVID-19, COVID-19 burden, and resilience. Psychological assistance is needed for frontline nursing staff to prevent insomnia symptoms and protect the mental health of frontline nursing staff after the end of China’s dynamic zero-COVID policy.

## Introduction

The COVID-19 pandemic is a significant challenge to global public health (1). It not only poses a significant threat to individual physical health but also causes severe mental health distress to people (2, 3). Due to the discovery that omicron variants are less pathogenic (4), the Chinese government officially ended the Dynamic Zero-COVID policy on January 8, 2023. Accordingly, it stopped all centralized quarantine and large-scale nucleic acid testing (5). However, Omicron variants have stronger transmissibility (6) and triggered a new wave of infections across China (7), resulting in a rapid surge in the number of infections in a short time (8). Like most countries worldwide, the COVID-19 pandemic severely affected the Chinese population and put enormous pressure on medical workers. After the Chinese government adjusted its response to COVID-19, there was an increase in patients and a shortage of medical resources within a certain period, which had a specific impact on the psychology of frontline medical staff (9). The nursing industry is prone to reduced melatonin levels and abnormal rhythm due to its unique characteristics of high stress, high load, and frequent night shifts (10). Rhythm disorder might lead to problems such as insomnia, difficulty concentrating, and even depression (11). It is well known that an appreciable segment of the frontline nursing staff has suffered from insomnia symptoms during the COVID-19 pandemic. For example, Vargas et al. (12) conducted a prospective cohort study and reported that insomnia symptoms may be associated with experiencing more chronic COVID-19 symptoms.

Insomnia symptoms refer to the subjective feeling of difficulty falling asleep, maintaining sleep for a long time, or being unable to achieve the effect of rest after waking up (13, 14). Insomnia symptoms are a complex and common problem affecting approximately 10–15% of adults (15, 16) worldwide. Insomnia symptoms are often associated with adverse physical and mental health outcomes, reduced quality of life, and even an increased risk of death, leading to significant global public health troubles (16–18). Research has found that insomnia symptoms have a high correlation with chronic diseases, and insomnia symptoms could bring significant direct and indirect costs (19). Insomnia symptoms not only seriously damage the physical and mental health of nursing staff but also reduce the efficiency and quality of nursing staff’s clinical nursing work and ultimately affect the safety of clinical nursing (20). Studies have shown that in the first 6 months of the COVID-19 pandemic, insomnia and PTSD symptoms increase dramatically. The results also show that insomnia symptoms seem to have been highly correlated with post-traumatic stress disorder (21).

During the COVID-19 pandemic, the incidence of insomnia symptoms in Chinese medical staff was 28.75%. Of these, approximately one-third to one-half of the frontline nursing staff reported insomnia symptoms (22). One study showed that the prevalence of insomnia symptoms among Chinese frontline nursing staff fighting COVID-19 in Wuhan was 52.8 (23). The incidence of insomnia symptoms among frontline medical staff in Shanghai, China, during the COVID-19 pandemic was 65.9%. ursing staff were more likely to have poor sleep quality and insomnia symptoms than doctors (24).

However, to our knowledge, there has been no previous study specifically on insomnia symptoms and related factors among frontline nursing staff in China after the end of China’s Dynamic Zero-COVID policy. Thus, it is necessary to investigate the insomnia symptoms status of frontline nursing staff and its influencing factors after the end of China’s Dynamic Zero-COVID policy. Therefore, the purposes of this study were to (1) examine the prevalence of insomnia symptoms among frontline nursing staff after the end of China’s Dynamic Zero-COVID policy; (2) examine the relationship between insomnia symptoms and PTSD, fear of COVID-19, COVID-19 burden and resilience among frontline nursing staff.

## Materials and methods

### Population and sample

The current study was conducted at Yijishan Hospital, Wannan Medical College, from March 2022 to August 2022. This study was a cross-sectional study among frontline nurses who have been infected with COVID-19. Patients voluntarily completed and returned the questionnaire and agreed to participate in the study. They could exit the survey at any time.

Participants in this study must meet the following inclusion criteria: (1) Frontline nursing staff during the study; (2) Understand the purpose and content of this study; (3) Have been infected with COVID-19; and (4) Willing to participate in the survey and sign the online electronic informed consent form.

### Data collection procedures

**Figure 1:**
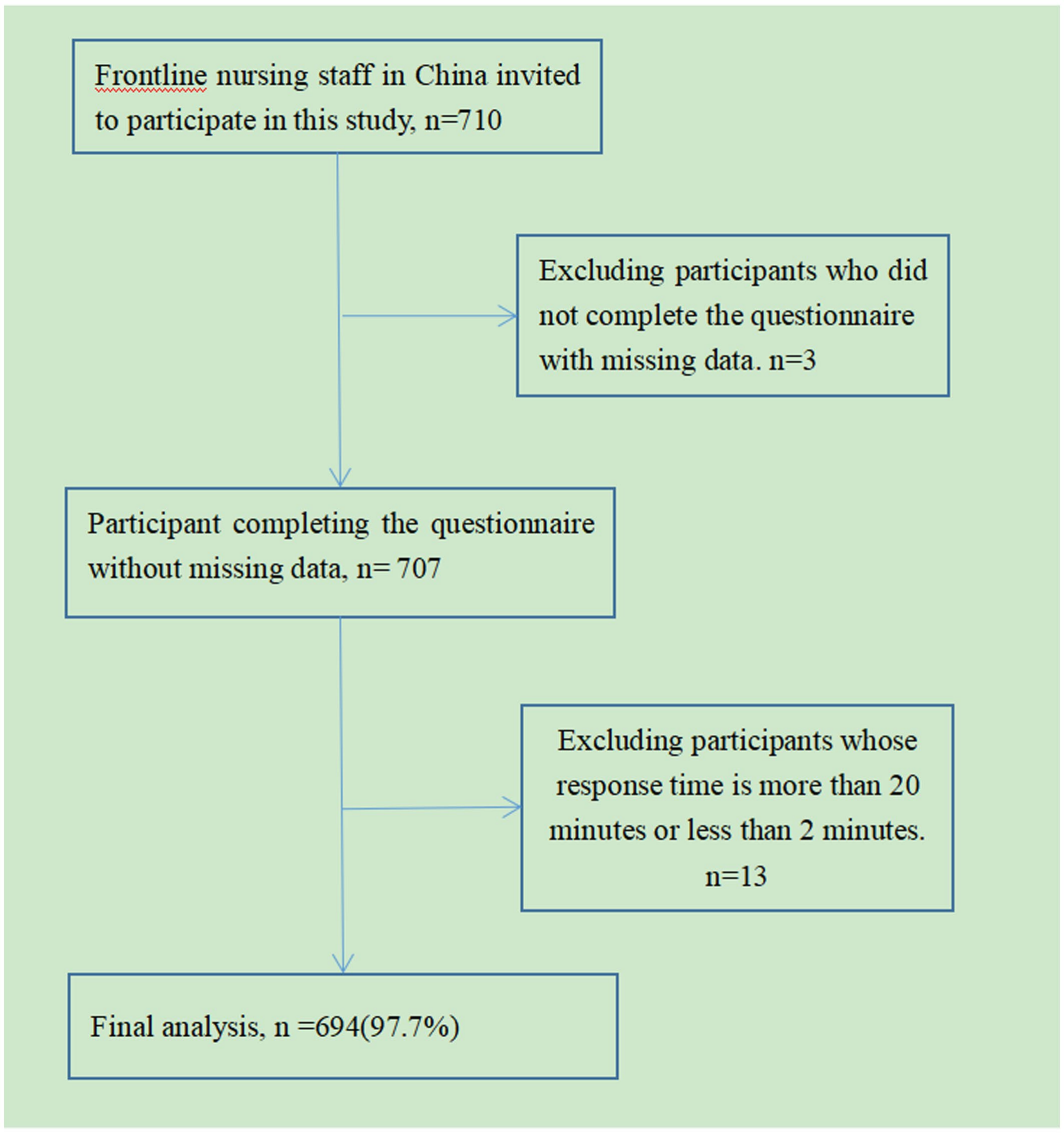
Sample selection process for this cross-sectional study.

The questionnaire was produced using the most widely used professional online survey website Wenjuanxing (URL: https://www.wjx.cn/). Members of the research team use QQ groups, WeChat groups and other chat tools to conduct outreach. To ensure data reliability, the same IP address can only be sent once. All questions must be answered prior to shipment. It would take about 5–10 min to complete all the questions. Nursing staff who completed the questionnaire forwarded the QR code and invited colleagues to participate. Participants carefully filled out the questionnaire and submitted it via smartphone or computer. A total of 710 electronic questionnaires were distributed, and after eliminating invalid questionnaires, 694 valid questionnaires were eventually included (details are shown in Figure 1).

### Instruments

#### Athens insomnia scale

The scale consists of eight items: the quality and duration of a night’s sleep and the respondent’s health status the next day. The first five points refer to falling asleep, night awakening, final awakening, total sleep time, and sleep quality, while the last three refer to functional deterioration the next day. Questions include “Recently, the delay in falling asleep (time after turning off the lights to falling asleep): no problem (less than 10 min), slight delay (10–30 min), significant delay (30–60 min), and severe delay or No sleep (more than 1 h)” and seven other questions. The score for each question ranges from 0 (no problem) to 3 (severe problem). The total score ranges from 0 to 24 points, the higher the score, the more severe the insomnia symptoms are, and the score ≥ 6 is considered as insomnia symptoms (25). The AIS has been reported to have good psychometric properties in the Chinese population (26). In the present study, the Cronbach’s alpha was 0.786.

#### PC-PTSD-5 Chinese version scale

The scale (27) consists of 5-items to assess PTSD experienced in the past month. Questions include “Have you ever had nightmares related to COVID-19 or have uncontrollable thoughts about COVID-19”? Each item is scored on a second level (0 = no; 1 = yes), with a total score ≥ 3 being classified as “PTSD symptoms.” The scale has been validated among family members of Chinese medical workers during COVID-19 pandemic (28). The Cronbach’s alpha was 0.915 in the present study.

#### Fear of COVID-19 scale

The scale (29) consists of 7 items, each item is rated on a 5-point Likert scale (1 = “strongly disagree,” 5 = “strongly agree”). Questions include “Thinking about COVID-19 makes me feel threatened.” The score ≥ 21 is considered as “fear of COVID-19.” The FCV-19S has been reported to have good psychometric properties in the Chinese population (29). In the present study, the Cronbach’s alpha was 0.86.

#### The 2-item Connor-Davidson resilience scale

The scale (30) consists of 2 items from items 1 and 5 of the CD-RISC-10 (31). Questions include “I can adapt when people and things around me change.” Each item is rated on a five-point Likert scale from 0 (never) to 4 (always). Research has proven that these two items best represent “adaptability” and “bouncing back” and have been proven to have good effectiveness in the Chinese population (32).

#### COVID-19 burden scale

COVID-19 burden was measured using the scale created by Nikunlaakso (33): “I have been afraid for my health because of the COVID-19 pandemic” and “My workload has increased because of the COVID-19 situation.” Measuring the COVID-19 burden on frontline nursing staff using two statements created by A: ① “I have been worried about my health due to the COVID-19 pandemic,” ② “COVID-19 pandemic has increased my workload,” with a response scale of yes/no. Respondents who answered two “yes” were classified as having a COVID-19 burden. The COVID-19 burden scale has been reported to have good psychometric properties in the Chinese population (27). In the present study, the Cronbach’s alpha was 0.82.

### Ethical considerations

This study was conducted in accordance with ethical guidelines and was approved by the Nursing Department of Yijishan Hospital, Wannan Medical College (No. 2023-02-01). Prior to their participation, all participants were fully informed about the study’s details and provided written informed consent. The study procedures adhered to the principles outlined in the Declaration of Helsinki.

### Statistical analysis

Statistical analysis of all data in this study was performed using SPSS 26.0 (IBM Corporation). *p* < 0.05(two-tailed) was the significant level. Demographic characteristics are presented with mean, standard deviation (SD), numbers, and percentages. Chi-square tests were used to compare the differences in categorical variables between the insomnia symptom group and the non-insomnia symptom group. Binary logistic regression analysis was used to analyze factors associated with insomnia symptoms among frontline nursing staff, and OR (odds ratio) and 95% CI (confidence interval) were calculated.

## Results

### Participant characteristics

Among 694 Chinese frontline nursing staff included in the data analysis, the respondents ranged from 18 to 60 years old, with the mean age being (25.56 ± 5.13) years old. 37 (5.3%) were male, and 657 (94.7%) were female. 159 (22.9%) were unmarried, 62 (8.9%) were married but childless, and 465 (67.0%) were married and had children. Besides, 36 (5.2%) were with unrated technical titles, 310 (44.7%) with Junior technical titles, 318 (45.8%) were with intermediate technical titles, and 30 (4.3%) were with high technical titles. Further socio-demographic information is displayed in Table 1.

**Table 1:** Participants’ demographic information (*N* = 694).

Variable	Category	Participants	Percentage (%)
Gender	Male	37	5.3
	Female	657	94.7
Age	<30	210	30.3
	30–39	379	54.6
	40–49	87	12.5
	≥50	18	2.6
Marital status	Unmarried	159	22.9
	Married but childless	62	8.9
	Married with children	465	67.0
	Other	8	1.2
Education	Technical secondary school	11	1.6
	Junior college	107	15.4
	Undergraduate	560	80.7
	Postgraduate and above	16	2.3
Technical title	Unrated	36	5.2
	Junior	310	44.7
	Intermediate	318	45.8
	High	30	4.3
Working years	<1 year	26	3.7
	1–2 years	27	3.9
	3–5 years	114	16.4
	6–10 years	180	25.9
	10–15 years	210	30.3
	>15 years	137	19.7
Hospital wards	Outpatient clinic	59	8.5
	Emergency medicine	20	2.9
	Blood purification center	95	13.7
	Respiratory medicine	41	5.9
	Geriatrics	10	1.4
	ICU	52	7.5
	Obstetrics	33	4.8
	Pediatrics	40	5.8
	Surgery	93	13.4
	Gynecology	53	7.6
	Others	198	28.5

### Factors associated with insomnia symptoms in the univariate analysis

In this study, the prevalence of insomnia symptoms among the frontline nursing staff was 74.5% (517/694). Insomnia symptoms are more severe among frontline nurses with the following characteristics: junior professional title or above, working for 1 year or more, burden of COVID-19, fear of COVID-19, and PTSD symptoms (*p* < 0.01, Table 2).

**Table 2:** Characteristics of the participants based on the presence of insomnia symptoms (*N* = 694).

	Non-insomnia symptoms (*n* = 177)	insomnia symptoms (*n* = 517)	χ^2^	*p*
Gender			0.367	0.544
Male	11 (29.7%)	27 (70.3%)		
Female	166 (25.3%)	490 (74.7%)		
Marital status			0.482	0.923
Unmarried	43 (27.0%)	116 (73.0%)		
Married but childless	14 (22.6%)	48 (77.4%)		
Married with children	118 (25.4%)	347 (74.6%)		
Other	2 (25.0%)	6 (75.0%)		
Age			4.476	0.214
<30	60 (28.6%)	150 (28.6%)		
30–39	87 (23.0%)	292 (77.0%)		
40–49	27 (31.0%)	60 (69.0%)		
≥50	3 (16.7%)	15 (83.3%)		
Education			1.541	0.673
Technical secondary school	4 (36.4%)	7 (63.6%)		
Junior college	30 (28.0%)	77 (72.0%)		
Undergraduate	138 (24.6%)	422 (75.4%)		
Postgraduate and above	5 (31.3%)	11 (68.8%)		
Technical title			14.952	<0.01
Unrated	19 (52.8%)	17 (47.2%)		
Junior	73 (23.5%)	237 (76.5%)		
Intermediate	78 (24.5%)	240 (75.5%)		
High	7 (23.3%)	23 (76.7%)		
Working years			19.113	<0.01
<1 year	15 (57.7%)	11 (42.3%)		
1–2 years	6 (22.2%)	21 (77.8%)		
3–5 years	31 (27.2%)	83 (72.8%)		
6–10 years	45 (25.0%)	135 (75.0%)		
10–15 years	41 (19.5%)	169 (80.5%)		
>15 years	39 (28.5%)	98 (71.5%)		
COVID-19 burden			49.370	<0.01
No	99 (41.6%)	139 (58.4%)		
Yes	78 (17.1%)	378 (82.9%)		
The fear of COVID-19			70.361	<0.01
No	104 (45.2%)	126 (54.8%)		
Yes	73 (15.7%)	391 (84.3%)		
The stage of COVID-19 infection			0.209	0.901
First infection	4 (22.2%)	14 (77.8%)		
Infected again	170 (25.5%)	496 (74.5%)		
About to recover	3 (30.0%)	7 (70.0%)		
Working while sick			17.472	<0.01
0 day	16 (45.7%)	19 (54.3%)		
1–3 days	64 (32.0%)	136 (68.0%)		
4–6 days	31 (24.2%)	97 (75.8%)		
≥7 days	66 (19.9%)	265 (80.1%)		
Resilience			30.214	<0.01
No	98 (19.8%)	398 (80.2%)		
Yes	79 (39.9%)	119 (60.1%)		
PTSD			49.297	<0.01
No	167 (32.3%)	350 (67.7%)		
Yes	10 (5.6%)	167 (94.4%)		

### Correlations between insomnia symptoms and relevant indicators

As shown in Table 3, the PTSD, the fear of COVID-19, working while sick, and the burden of COVID-19 of frontline nursing staff had positive correlations among their insomnia symptoms (*p* < 0.01). In contrast, resilience had a negative correlation (*p* < 0.01).

**Table 3:** Correlation between insomnia symptoms and relevant indicators in frontline nursing staff.

Factors	*r*	*p* value
PTSD	0.462	<0.001**
The Fear of COVID-19	0.455	<0.001**
COVID-19 burden	0.323	<0.001**
Resilience	−0.217	<0.001**

** *p* < 0.01.

### Binary analysis factors associated with insomnia symptoms

In the binary logistic regression analysis, put independent variables (*p* < 0.05) and dependent variables (grouping, 0 = non-insomnia symptoms group, 1 = insomnia symptoms group) into the model. Factors affecting insomnia symptoms in frontline nursing staff are shown in Table 4. Insomnia symptoms are more severe among frontline nursing staff with PTSD (OR = 4.693, 95% CI 2.325–9.473). The more severe the fear of COVID-19, the higher the insomnia symptoms score (OR = 2.307, 95% CI 1.541–3.452). COVID-19 burden increased the risk of insomnia symptoms (OR = 2.093, 95% CI 1.407–3.114). Compared with ungraded frontline nursing staff, frontline nursing staff with high professional titles showed more symptoms of insomnia symptoms (OR = 3.947, 95% CI 1.769–8.807; OR = 3.626, 95% CI 1.630–8.067; OR = 5.256, 95% CI 1.550–17.823). Resilience was a protective factor for insomnia symptoms (OR = 0.478, 95% CI 0.321–0.713).

**Table 4:** Binary logistic regression analysis of factors associated with insomnia symptoms.

Indices	β	Wald	*p* value	OR	95% CI
The Fear of COVID-19 (No = 0, Yes = 1)	0.836	16.504	<0.001	2.307	1.541–3.452
PTSD (No = 0, Yes = 1)	1.546	18.615	<0.001	4.693	2.325–9.473
COVID-19 burden (No = 0, Yes = 1)	0.739	13.295	<0.001	2.093	1.407–3.114
Technical title (Junior = 1)	1.373	11.247	0.001	3.947	1.769–8.807
Technical title (Intermediate = 2)	1.288	9.971	0.002	3.626	1.630–8.067
Technical title (High = 3)	1.659	7.094	0.008	5.256	1.550–17.823
Resilience (No = 0, Yes = 1)	−0.738	13.107	<0.001	0.478	0.321–0.713

## Discussion

During the COVID-19 pandemic, the insomnia symptoms of frontline nursing staff have attracted increasing attention from domestic and foreign scientists. To our knowledge, this is the first study investigating the prevalence of insomnia symptoms and influencing factors among frontline nursing staff in China after the ending of China’s dynamic zero-COVID policy. In this study, we found that the prevalence of insomnia symptoms based on a total Athens Insomnia Scale score of ≥6 was 74.5% among Chinese frontline nursing staff, higher than the annual incidence of insomnia symptoms in adults (34), and the ratio of insomnia symptoms among healthcare workers during COVID-19 pandemic (35). This difference may be associated with the surge in COVID-19 cases in China after the ending of China’s dynamic zero-COVID policy. Because most frontline nursing staff had never experienced such a prolonged and severe epidemic, leading to increased work pressure and resulting in insomnia symptoms. This study did not find the impact of the disease itself (the stage of COVID-19 infection, working while sick) on sleep. This may be because everyone is infected, so the impact of the disease itself already exists, so the difference is narrowed.

After the ending of China’s dynamic zero-COVID policy in December 2022, the frequency and number of hospitalizations due to Covid-19 increased dramatically (36). During the epidemic of COVID-19, nursing staff, as an important part of the anti-epidemic staff, bear various negative effects caused by COVID-19. COVID-19 can cause distress for nursing staff working in hospitals, firstly because of the increased shift hours and disrupted biological rhythms during quarantine, which can lead to insomnia symptoms (37), and secondly because of the increased risk of infection, isolation, and loss of social support, friends and relatives, which can impair their resilience (38). During the COVID-19 pandemic, frontline nursing staff have been caring for infected patients, making them more vulnerable to the psychological consequences. Studies have found that health care workers caring for COVID-19 patients were at higher risk of insomnia symptoms (39, 40), burnout, and post-traumatic stress disorder (PTSD) (27) due to excessive work pressure. Among all healthcare professionals, frontline nursing staff are particularly susceptible to sleep disorders during the COVID-19 pandemic (41). This implies that after the ending of China’s dynamic zero-COVID policy, the risk of insomnia symptoms among frontline nursing staff caregivers under long-term physical and psychological stress increases significantly.

Our result is consistent with a previous study that the fear of COVID-19 is associated with insomnia symptoms. Study have shown that there was a significant relationship between the level of fear of COVID-19 and insomnia symptoms (42). Research has shown that the fear of COVID-19 among Chinese mental health professionals increased after the end of the dynamic zero-COVID policy (43). More than one-third of fire service recruits feared COVID-19 after the end of China’s dynamic zero-COVID policy (44), and “Sleep difficulties caused by worry about COVID-19” was the central symptom in the network structure of COVID-19 fear. A study in the United Kingdom found that more than half of mental health professionals experienced insomnia symptoms during the COVID-19 pandemic (45). Studies have found that mass media might have exacerbated the fear of COVID-19 due to their over-reporting of inaccurate news on social media, such as the “coronavirus infodemic,” which could exacerbate people’s psychological distress (43). Furthermore, avoiding excessive fear of COVID-19 may be beneficial in alleviating insomnia symptoms among frontline nurses.

We found that the higher the PTSD score, the more likely the frontline nursing staff were to suffer from insomnia symptoms. This finding could be explained by the following fact: PTSD symptom severity is significantly correlated with insomnia symptoms, and the relationship between PTSD severity and insomnia symptoms seems to be mediated entirely by nightmare severity (46). It has been argued that, for individuals, the experience of the COVID-19 pandemic can be viewed as a mass traumatic event, and PTSD may develop following trauma (47). The surge in hospital admissions due to COVID-19 has increased the workload on nursing staff. One study found that 44.1% of SARS patients developed PTSD symptoms within 2 to 46 months after discharge (48). Also, the research found that PTSD caused shortened sleeping time and poorer quality of life in frontline nursing staff exposed to COVID-19 (49). These results suggested that future attention should be paid to the PTSD symptoms among frontline nursing staff, and effective measures to alleviate these symptoms should be taken.

Our study found that nursing staff with higher professional titles had more severe insomnia symptoms than nursing staff who were unrated. Nursing staff’s professional title is the basis for their career planning, directly affecting their participation in learning, training, and professional title promotion in clinical work. Nursing staff with higher professional titles usually have rich clinical experience and professional knowledge and hold corresponding management positions. In addition to busy nursing work, they also need to deal with more complex medical management and organizational issues and assume more responsibilities and decision-making, which may also cause them to face tremendous pressure. At the same time, nursing staff with higher professional titles tend to be older, and studies have found that older people are more likely to suffer from insomnia symptoms (50).

Consistent with a previous study (49), this study found resilience was a protective factor in insomnia symptoms among frontline nursing staff. Resilience is the ability to help individuals cope with adversity and recover quickly from stressful experiences. It can help individuals cope with situations such as crises, trauma, and adversity (50). Poor resilience was one of the essential factors leading to insomnia (51). A study reported that psychological resilience was a critical protective factor in reducing COVID-19 fear (52). During the COVID-19 pandemic, resilience can help healthcare workers protect themselves (53), help them channel and cope with the stress caused by the pandemic, and thus better respond to disasters and survive the crisis (54).

### Limitations

This study may have the following limitations: (1) Cross-sectional design: The cross-sectional design adopted in this study limits the ability to determine the causal relationship between insomnia symptoms and related factors. (2) Self-report measures: This study used self-reported insomnia symptoms by frontline nursing staff, which may introduce memory bias and subjective interpretation. (3) Regional limitations: This study was only conducted in one region of China, and the results may not fully represent frontline nursing staff in other countries or regions.

## Conclusion

In summary, after the ending of China’s dynamic zero-COVID policy, frontline nursing staff showed severe symptoms of insomnia symptoms. PTSD, COVID-19 fear, and COVID-19 burden were all significantly associated with insomnia symptoms, while psychological resilience was a protective factor for insomnia symptoms. Health committees and policymakers should pay attention to the impact of the above factors on insomnia symptoms among frontline nursing staff and actively develop effective intervention measures to reduce insomnia symptoms among frontline nursing staff.

## Data availability statement

The raw data supporting the conclusions of this article will be made available by the authors, without undue reservation.

## Ethics statement

The studies involving humans were approved by this study was conducted in accordance with ethical guidelines and the Nursing Department of Yijishan Hospital, Wannan Medical College (No. 2023-02-01). The studies were conducted in accordance with the local legislation and institutional requirements. The participants provided their written informed consent to participate in this study. Written informed consent was obtained from the individual(s) for the publication of any potentially identifiable images or data included in this article.

## Author contributions

MZ: Conceptualization, Formal analysis, Investigation, Resources, Supervision, Writing – original draft, Writing – review & editing. CC: Investigation, Visualization, Writing – original draft, Writing – review & editing. QL: Data curation, Formal analysis, Software, Writing – original draft, Writing – review & editing. NZ: Data curation, Investigation, Methodology, Visualization, Writing – original draft. ZZ: Investigation, Methodology, Supervision, Writing – review & editing. XT: Formal analysis, Investigation, Methodology, Supervision, Writing – review & editing. BX: Conceptualization, Investigation, Methodology, Software, Supervision, Writing – original draft, Writing – review & editing. HL: Data curation, Investigation, Methodology, Software, Visualization, Writing – original draft, Writing – review & editing.
